# Diagnostic Performance of an *Aspergillus*-Specific Nested PCR Assay in Cerebrospinal Fluid Samples of Immunocompromised Patients for Detection of Central Nervous System Aspergillosis

**DOI:** 10.1371/journal.pone.0056706

**Published:** 2013-02-22

**Authors:** Mark Reinwald, Dieter Buchheidt, Margit Hummel, Matthias Duerken, Hartmut Bertz, Rainer Schwerdtfeger, Stefan Reuter, Michael G. Kiehl, Manuel Barreto-Miranda, Wolf-Karsten Hofmann, Birgit Spiess

**Affiliations:** 1 Department of Hematology and Oncology, Mannheim University Hospital, University of Heidelberg, Mannheim, Germany; 2 Department of Pediatrics, Mannheim University Hospital, University of Heidelberg, Mannheim, Germany; 3 Freiburg University Hospital, University of Heidelberg, Mannheim, Germany; 4 Bone Marrow Transplantation Center Wiesbaden, Wiesbaden, Germany; 5 Ulm University Hospital, University of Ulm, Ulm, Germany; 6 Frankfurt/Oder General Hospital, Frankfurt (Oder), Germany; University of Toronto, Canada

## Abstract

Central nervous system (CNS) invasive aspergillosis (IA) is a fatal complication in immunocompromised patients. Confirming the diagnosis is rarely accomplished as invasive procedures are impaired by neutropenia and low platelet count. Cerebrospinal fluid (CSF) cultures or galactomannan (GM) regularly yield negative results thus suggesting the need for improving diagnostic procedures. Therefore the performance of an established *Aspergillus*-specific nested polymerase chain reaction assay (PCR) in CSF samples of immunocompromised patients with suspicion of CNS IA was evaluated. We identified 113 CSF samples from 55 immunocompromised patients for whom CNS aspergillosis was suspected. Of these patients 8/55 were identified as having proven/probable CNS IA while the remaining 47 patients were classified as having either possible (n = 22) or no CNS IA (n = 25). PCR positivity in CSF was observed for 8/8 proven/probable, in 4/22 possible CNS IA patients and in 2/25 NoIA patients yielding sensitivity and specificity values of 1.0 (95% CI 0.68–1) and 0.93 (95% CI 0.77–0.98) and a positive likelihood ratio of 14 and negative likelihood ratio of 0.0, respectively, thus resulting in a diagnostic odds ratio of ∞. The retrospective analysis of CSF samples from patients with suspected CNS IA yielded a high sensitivity of the nested PCR assay. PCR testing of CSF samples is recommended for patients for whom CNS IA is suspected, especially for those whose clinical condition does not allow invasive procedures as a positive PCR result makes the presence of CNS IA in that patient population highly likely.

## Introduction

Cerebral aspergillosis is a frequent and often lethal complication of disseminated invasive Aspergillosis (IA). [1] Mortality rates were found to be as high as 88% in a meta-analysis which reported on 1941 patients and encompassed a timeframe from 1995 to 1999. [2] Despite improved radiological techniques, the introduction of voriconazole and the addition of neurosurgical treatment procedures which have significantly improved the outcome [3], the response rate in severely immunocompromised patients is still poor with hematopoietic stem cell transplantation (HSCT) recipients showing responses in less than 15%. [4].

Diagnostic procedures are still insufficient. Improvements have been made by introducing magnetic resonance imaging (MRI) into the armamentarium, [5] however radiomorphological signs are suggestive, but not specific. Thus stereotactical biopsies with histology and microbiological culture procedures still remain the gold standard in obtaining definite proof of cerebral IA. However, especially in the hematological patient population underlying low platelet count or neutropenia precludes these procedures or significantly increases risk of complications. Therefore there is an urgent need for improving the diagnostic certainty by obtaining microbiologic evidence of cerebral IA with less invasive procedures.

Cerebrospinal fluid (CSF), which can usually be obtained with low risk in patients with low platelet count, would be the obvious choice. However CSF cultures are usually negative [6] and cell count, glucose or protein concentration are not specific. Nevertheless it is usually performed to rule out other infectious causes in routine clinical practice. The clinical evidence of surrogate parameters like galactomannan (GM) in CSF for diagnosis of cerebral IA is scarce and provided as case-series with small numbers of patients. [7], [8], [9] Despite this small numbers (n = 5), CSF GM is included as microbiological evidence in the European Organization for Research and Treatment of Cancer Invasive Fungal Infections Cooperative Group and the National Institute of Allergy and Infectious Diseases Mycoses Study Group (EORTC/MSG) [10] invasive fungal infections (IFI) consensus criteria and mentioned in the recent European Conference on Infections in Leukemia (ECIL-3) recommendations. [11].

Polymerase chain reaction (PCR) assays have shown to be another promising diagnostic approach, however, up to now they are not part of the EORTC/MSG IFI consensus criteria, as there is a variety of methods and lack of standardization. Although several groups have reported on the performance of PCR for diagnosing IA in blood [12] or BAL, [13], [14] its use in CSF for diagnosing cerebral IA has rarely been reported. Our group published on the successful use of a nested *Aspergillus*-specific PCR assay in CSF samples of four proven/probable cerebral aspergillosis patients [15], underlining the potential of this molecular diagnostic tool in this clinical setting. Recently, another group observed positive Aspergillus-specific PCR signals in CSF using real-time PCR in 6/38 patients with heterogeneous underlying conditions. In that study, two consecutive PCR results defined the diagnosis, however CSF cultures for Aspergillus were negative and biopsy results were not reported [16]. Kami et al. observed a better sensitivity of CSF PCR compared to CSF GM in 5 probable CNS IA patients; [17] all other remaining publications are single case reports. Additionally, in an animal model of experimentally-induced cerebral aspergillosis a high sensitivity of CSF PCR for diagnosing cerebral IA was observed. [18].

In order to further elucidate the performance of an *Aspergillus* specific PCR assay in CSF for diagnosing cerebral IA we performed this multicenter trial.

## Patients and Methods

### Patients

CSF samples submitted to the scientific laboratory of the 3^rd^ Medical Department of the Mannheim University Hospital for diagnosing cerebral IA between February of 1999 and May 2011 were analyzed to elucidate PCR performance in CSF.

Immunocompromised patients at high risk for fungal infections were included in this retrospective analysis. Written informed consent of patients or legal representatives had been acquired prior to CSF sampling and analysis was done according to Good Clinical Practice (GCP) guidelines as well as in concordance with the Declaration of Helsinki. The study was approved by the local Ethics Committee (Ethics Committee of the Faculty of Medicine, University of Heidelberg, Germany; Reference Number 2007-240N-MA). The trial was registered by ClinicalTrials.gov (Identifier: NCT01617759).

### Patient Characteristics

One hundred thirteen CSF samples from 55 immunocompromised pediatric and adult patients at high risk for IA were obtained between February of 1999 and May of 2011 and analysed by an *Aspergillus*-specific nested PCR assay described previously [19]; for patients characteristics see Table 1 and 2.

**Table 1:** Patient characteristics.

Characteristics	Proven/Probable CNS IA*(n = 8)	Possible/CNS IA*(n = 22)	No CNS IA (n = 25)	
Age (median, range)	38 (4–82)	42 (8–68)		55 (0–73)
Gender (female/male)	4/4	11/11		14/11
Underlying disease				
AML	1	8		3
ALL	4	4		7
NHL	2	4		10
AIHA	0	0		1
MPN	0	1		0
MDS	0	1		0
Aplastic Anemia	0	1		0
CVID	0	1		0
Solid Tumor	0	0		1
Other	1	2		3
Among them patients after/with				
Allo-HSCT	3	9		2
Auto-HSCT	0	1		0
* HIV* infection	0	1		0

* according to 2008 EORTC/MSG Criteria modified by Schwartz et al.

AML: acute myeloid leukemia; ALL acute lymphoblastic leukemia; NHL Non-Hodgkińs-Lymphoma; MDS: Myelodysplastic Syndrome; MPN: myeloproliferative neoplasia; Allo-HSCT: allogeneic hematopoietic stem cell transplantation; Auto-HSCT: autologous hematopoietic stem cell transplantation; AIHA: Autoimmunehemolytic anemia; CVID : common variable immunodeficiency syndrome; *other*: Primary chronic polyarthritis, HIV infection, sarcoidosis, miliary tuberculosis, bacterial meningitis.

**Table 2:** Clinical data for proven/probable patients and possible/NoIA patients positive for Aspergillus PCR.

Patient ID	Underlying disease	Definition of CNS IA	Other Sites of IA	Suspected IA outside CNS according to EORTC/MSG	Neutropenia	SCT	GvHD	Steroids	CNS Radiomorphology	CNS results	CSF GM	Origin of Aspergillus detection defining probability&	Antifungal treatment prior to CSF sampling
1	ALL	Proven	None	None	None	Allo	Yes	Yes	Abscess-like lesion with ring enhancement	CSF culture positive	n.d.	CSF culture positive	L-AMB+Caspofungin
2	ALL	Proven	Lung, Spleen, Liver	Lung (probable)	Yes	None	n.a.	Yes	Multiple intracerebral abscesses	CSF culture positive	Negative	CSF culture positive	Voriconazole
3	NHL	proven	Sinusitis	Sinusitis (possible)	Yes	None	n.a.	Yes	Abscess-like lesion with ring enhancement	CNS resection positive	Negative	CNS resection positive	none
4	NHL	Proven	Sinusitis	Sinusitis (possible)	Yes	None	n.a.	Yes#	Abscess-like lesion with ring enhancement	CSF culture positive	n.d.	CSF culture positive	Voriconazole
5	PCP	Proven	Sinusitis, Orbital infiltration	Sinusitis, Orbital (possible)	None	None	n.a.	Yes$	Progressive abscess-like Lesion with penetration into Sinus and Orbita	CNS biopsy positive	n.d.	CSF biopsy positive	Voriconazole
6	AML	Probable	Lung	Lung (probable)	Yes	Allo	None	None	Multiple intracerebral abscesses	CNS culture negative	n.d.	Typical radiomorphology+Allo-SCT +positive Sputum culture	none
7	ALL	Probable	Lung	Lung (possible)	Yes	None	n.a.	Yes	Solitary abscess, in the course of the disease ventriculitis	CSF GM positive	Positive	CSF GM positive	Voriconazole+L-AMB+Caspofungin
8	ALL	Probable	Lung	Lung (possible)	Yes	Allo	None	Yes	Multiple intracerebral abscesses	CSF GM positive	Positive	CSF GM positive	Caspofungin, Voriconazole
9	ALL	Possible	Sinusitis	Sinusitis (possible)	Yes	None	n.a.	Yes	Pansinusitis with infiltration, meningeal enhancement	Negative	n.d.	n.a.	none
10	CLL	Possible	None	None	Yes	None	n.a.	Yes	Periventricular enhancement compatible with meningoencephalitis	Negative	n.d.	n.a.	None
11	CVID	Possible	Sinusitis, Lung	Sinusitis (possible) Lung (probable)	None	None	n.a.	None	Multiple intracerebral abscesses	Negative	BAL GM 5.9 OD	BAL GM positive, however no positive culture	Voriconazole
12	ALL	Possible	Lung	Lung (possible)	Yes	None	n.a.	Yes	Meningeal enhancement	Negative	Negative	n.a.	None
13	AML	NoIA	Hepatic Lesions	n.a.	None	Allo	None	Yes	None	Negative	Negative	n.a.	Voriconazole
14	NHL	NoIA	None	n.a.	None	None	None	Yes	None	Negative	n.d.	n.a.	None

L-AMB = liposomal amphotericin B; ALL = acute lymphoblastic leukemia; NHL = non-Hodgkin-lymphoma; PCP = primary chronic polyarthritis; CLL = chronic lymphocytic leukemia; AML = acute myeloid leukemia; SCT = hematopoietic stem cell transplantation; GvHD = graft-versus-host-disease; BAL = bronchoalveolar lavage; GM = galactomannan; n.d. = not done.

& according to Schwartz et al. Blood 2005 [3].

$ chronic steroid treatment for primary chronic polyarthritis.

# chronic steroid treatment for autoimmunehemolytic anemia induced by NHL.

CSF samples were referred from the University Hospitals of Mannheim, Jena, Freiburg, Ulm, Bochum, and Düsseldorf; Bone Marrow Transplantation Center Wiesbaden; General Hospitals of Frankfurt/Oder, Erfurt and Schwedt; all in Germany.

### Classification of Cerebral Aspergillosis

The patients were classified according to the proposed case definitions for CNS IA by Schwartz et al.: [3] In proven CNS aspergillosis there was cultural or histologic evidence for *Aspergillus* in cerebrospinal fluid or brain biopsy specimens. Patients classified to have proven CNS aspergillosis without positive fungal cultures from the CNS required a positive histologic or cytologic specimen from brain/CNS and a positive culture with growth of *Aspergillus* from other sterile body sites or bronchoalveolar lavage. Patients defined as having probable CNS IA had radiologic signs of CNS infection in cranial computed tomographic (cCT) or magnetic resonance imaging (MRI) scans plus a proven *Aspergillus* infection at body sites outside the CNS. Proven aspergillosis outside the CNS was classified according to the latest EORTC/MSG criteria. [10] Furthermore, highly immunocompromised patients (either allogeneic stem cell transplantation or with neutropenic hematological disease) with imaging suggesting invasive aspergillosis plus signs and symptoms of a CNS infection were also classified as probable CNS aspergillosis if they had a positive Aspergillus culture from a nonsterile site or an adequate antigen result from cerebrospinal fluid. Patients defined to have possible CNS IA had compatible imaging and host factors but no microbiologic criterion for probable CNS IA, while the NoIA group consisted of patients for whom CNS IA was highly unlikely based on the lack of intensive immunosuppression (insufficient host factor criteria) or without compatible radiological findings.

### Radiological Diagnostics

All patients received either cMRI or cCT prior to CSF sampling performed according to standardized techniques. Radiological diagnostic scan results were analysed by experienced radiologists at the time of imaging in a non-blinded fashion. Typical lesions for CNS IA in MRI consisted of abscesses, mass lesions or infarction-like lesions, unlikely for other infectious causes or bleeding.

### CSF Sampling

CSF sampling was performed as clinically indicated; reasons for CSF sampling were suspected CNS infection or suspicion of meningeal involvement or CNS manifestation of the underlying disease. CSF sampling was not performed solely for study reasons. Vials of 1 to 2 ml of CSF were shipped immediately with delivery time of less than 24 hours and the specimens were sent at ambient temperature. The diagnostic work-up was usually performed according to the guidelines of the Infectious Diseases Working Party (AGIHO) of the German Society of Hematology and Oncology (DGHO). [20], [21] Typical CSF volumes were between 1–2 mL. Median cell count in the proven/probable CNS IA patients was 3/µl (range 1–266/µl), 4/µ (range 0–777/µl) for the possible patients and 1/µl for the NoIA patients (range 0–524).

### DNA Preparation and PCR Analysis

Total DNA was extracted from 1.0 mL of CSF samples and processed by an experienced technical assistant uninformed of clinical data according to the DNA extraction and nested PCR protocol published by Skladny et al. [19] and modified for CSF by Hummel et al. [15].

Briefly, in contrast to DNA extraction from other clinical samples, DNA from CSF was extracted from the whole sample (1.0 to 2 ml) instead of from the cell pellet. Purification of DNA was performed by phenol-chloroform extraction. 100 ng of total DNA was used as the template per 25-ml PCR mixture. In the nested two-step PCR technique, two pairs of oligonucleotide primers (AFU 7S and AFU 7AS for the first step and AFU 5S and AFU 5AS for the second step), derived from sequences of the *A. fumigatus* 18S rRNA gene (GenBank accession no. AB008401) and specific for *Aspergillus* species, were used (Skladny et al. [19]). A 138-bp PCR fragment encoded by the human glucose-6-phosphate dehydrogenase gene (GenBank accession no. X55448) was amplified by primers G6PD1S and 1AS in each clinical sample as an internal control. All PCR reactions were performed as duplicates. The sensitivity of this nested PCR assay is 1 to 5 CFU per ml of CSF.

PCR amplification was done as follows: The standard PCR mixture contained 0.5 U of *Taq* DNA polymerase, 6.25 nmol of the deoxynucleoside triphosphates, 10 pmol of primer (first step, primer AFU7S-AFU7AS; second step, primer AFU5S-AFU5AS). PCR was performed using the following conditions: For the first PCR, 2 min at 94°C and then 23 cycles of 40 s at 94°C, 1 min at 65°C, and 1 min at 72°C with a terminal step of 5 min at 72°C and then the mixture was held at 4°C; for the second PCR, 2 min at 94°C and then 35 cycles of 40 s at 94°C, 1 min at 65°C, and 1 min at 72°C, with a terminal step of 5 min at 72°C, and then the mixture was held at 4°C. For the second PCR, approximately 1 to 2 ml of the first-round PCR product was used. The PCR products were separated by 2.5% agarose gel electrophoresis, stained with ethidium bromide, and visualized with UV light. Control samples included all the constituents in the reaction mixture except genomic DNA. Diluted samples of *A. fumigatus* were used as positive controls, whereas DNA from the human cell line T47D was used as a negative control. The PCR assay used in this study has been shown to detect a minimum of seven *Aspergillus* species: *A. fumigatus, A. flavus, A. niger, A. terreus, A. clavatus, A. versicolor* and *A. nidulans*), whereas the PCR assay does not detect other fungal, bacterial or human DNA. The amplicon has been sequenced in the methodological establishment of this assay and has been shown to match the corresponding GenBank sequence. [19].

### Statistical Analysis

Sensitivity rates, specificity rates, positive and negative likelihood ratios, diagnostic odds ratio as well were performed using GraphPad Prism for Windows 5.0 (GraphPad Software, La Jolla, CA, USA) and Microsoft Excel 2010 (Microsoft Software, Redmond, USA). Statistical analysis for comparing PCR positivity was performed using Wilcoxon signed rank test, comparison of CSF cell count was performed using the Kruskal-Wallis-test and Mann-Whitney-U-test. P values of less than.05 were considered statistically significant differences.

## Results

According to the aforementioned criteria, five patients were classified as proven, three patients as having probable cerebral aspergillosis, as well as 22 patients having possible CNS IA, the remaining 25 patients did not fulfil the EORTC/MSG criteria for invasive fungal disease (modified by Schwartz et al [3] for CNS IA).

For determination of PCR performance all proven/probable patients (n = 8) were evaluated while the remaining noIA patients (n = 25) were used as the control population, the possible patients (n = 22) were excluded from the analysis of PCR performance.

Positive PCR signals were detected in 22 samples from 14 patients. Of these, 5 patients were classified as proven, 3 as probable and 4 as possible CNS IA; two patients having no CNS IA were found to be PCR positive.

PCR positivity was significantly higher in patients classified as having proven/probable CNS aspergillosis compared to NoIA patients (p<0.006).

There was no statistical difference in CSF cell count between proven/probable CNS IA patients, possible CNS IA patients and no NoIA patients. When comparing the CSF cell count in the PCR-positive patients (n = 14) compared to the PCR negative patients (n = 41) no statistical difference was observed.

### Diagnostic Performance of CSF PCR in Proven/Probable (n = 8) vs. NoIA Cases (n = 25)

For patients with proven cerebral aspergillosis (n = 5) as well as for the 3 patients with probable cerebral aspergillosis, PCR was found to be positive in all cases (8/8). In the other patients positive PCR signals were observed in 6/47 cases. Of these, four were classified as having possible cerebral IA while the remaining two were classified as having NoIA. Of these 6 cases 4 had imaging results compatible with CNS IA, two had negative imaging results. In these 6 patients no biopsies or autopsies were performed, CSF culture was negative and in two of these patients GM CSF was negative.

Observed test performance parameters for PCR (comparing proven/probable cases vs. NoIA patients) yielded sensitivity and specificity values of 1.0 (95% CI, 0.68–1.0) and 0.93 (95% CI, 0.77–0.98) respectively, with a positive likelihood ratio (PLR) of 14, a negative likelihood ratio (NLR) of 0 and a diagnostic odds ratio of >200 (∞). (Table 3).

**Table 3:** Diagnostic performance of CSF PCR.

Case definition vs NoIA cases	Sensitivity (95% CI)	Specificity (95% CI)	PLR	NLR	DOR
proven & probable (n = 8)	1.0 (0.61–1.0)	0.93 (0.77–0.98)	12	0	>200

PLR = Positive likelihood ratio; NLR = Negative likelihood ratio; DOR = Diagnostic odds ratio.

## Discussion

We systematically investigated the performance of an *Aspergillus* specific PCR assay in CSF samples of immunocompromised patients for evaluating its ability to diagnose CNS IA. All patients who suffered from CNS aspergillosis as defined by the strict criteria by Schwartz et al. ^3^ showed a positive PCR signal in CSF. Although this conclusion is based on a limited number of patients for whom CNS aspergillosis is definitely present, we found its performance promising. Despite these seemingly small numbers, our study reports the largest number of proven/probable CNS IA cases up to now in terms of evaluating diagnostic tests or surrogate parameter performance in CSF samples for CNS aspergillosis.

One of the major hurdles when investigating CNS aspergillosis is that a definite proof can usually only be obtained via neurosurgical procedures, despite the fact that it represents the third most frequent manifestation of disseminated IA [1] and its incidence has been reported to be as high as 21% in patients with IA in an autopsy-based study [22]. Therefore, obtaining an ample number of cases for evaluating a diagnostic tool in that patient population seems rather difficult and explains the lack of larger studies on that particular topic. Although it might be suspected in immunocompromised febrile patients with focal lesions in cMRI or cCT refractory to broad-spectrum antibiotics, particularly in HSCT recipients, radiomorphologic results of IA can resemble infections caused by other organisms such as *Cryptococcus neoformans*, *Candida spp*., *Nocardia* spp., toxoplasmosis or tuberculosis. [23] Furthermore, hematologic patients or HSCT recipients often present with clinical conditions/comorbidities which do not allow invasive neurosurgical procedures, therefore antifungal therapy is usually applied in a preemptive approach.

CSF testing has advantages, as it can be safely performed in patients with low platelet count [24]. It would therefore be applicable in the majority of hematologic patients, even in those not deemed eligible for invasive procedures. It has its traditional place value in CNS infections as other infections (nocardiosis, cryptococcosis) may be ruled out. For diagnosing CNS IA CSF GM has been included in the EORTC/MSG Consensus criteria and is therefore recommended for patients with suspected CNS IA. The scientific background for this recommendation however is rather scarce: Viscoli et al. reported in 2002 on five patients with suspected CNS IA; they found GM significantly elevated in CSF of these 5 patients compared to 16 control CSF samples. Of these patients only 3 were autopsy-proven, the other 2 had a nasal swab positive for *Aspergillus* culture and no biopsies of the lesions were performed. [8] In another report, 26 CSF samples from one patient with proven *Aspergillus* meningitis were evaluated for GM, the authors found it to be significantly elevated and its CSF level decreased with clinical and radiological improvement. [7] Additionally performed *Aspergillus*-specific PCR was found to yield positive signals in several CSF samples. Recently, CSF was analyzed for non-cryptococcal fungal meningitis in a study which included immunocompromised and trauma patients [16] using real-time PCR with primers, specific for both *Aspergillus* species and *Candida*. Altogether 6 positive *Aspergillus* PCR results were observed. CSF cultures were negative however, biopsies or CSF GM were not reported, so that, according to the EORTC Criteria modified by Schwartz, the definition for proven/probable CNS IA was not met, as a positive PCR signal is not accepted as microbiologic evidence.

Our study, despite being based on only 8 patients with proven/probable CNS aspergillosis shows very good performance of *Aspergillus* PCR for diagnosing CNS IA in CSF, with at least one sample from each patient showing a positive result. This is in line with the observation made by Kami et al, who reported about PCR positivity in 5 patients with probable CNS IA case definition [3] They additionally compared its performance with CSF GM and found that GM performed slightly worse with positive results in 4/5 samples. Contrary to the control samples in that publication, we found positive PCR signals in the other patients (6/47; 4 possible IA samples, two No CNS IA samples) which may be attributed to the high sensitivity of our nested PCR approach. However, none of these six patients had either autopsy or biopsy performed, all had negative CSF culture results and in addition two out of six pts had a negative CSF GM, therefore “upgrading” these patients to probable IA was not possible. This represents a rather common problem when evaluating diagnostic tests in IA; it is not clear if patients with only possible IA might nevertheless still be suffering from the infection, but lack microbiological evidence, as the standard methods defining positivity (e.g. culture) lack sensitivity. Thus, comparing our proven/probable study population solely to the cases classified as having NoIA specificity shows very good results.

We recognize that our results about *Aspergillus*-specific PCR performance are based on a small cohort of patients, albeit our data represents the biggest series of CSF analysis of surrogate parameter performance for CNS aspergillosis to date.

In conclusion, PCR testing of CSF is promising for patients in whom CNS aspergillosis is suspected, especially for those whose clinical condition does not allow invasive diagnostic procedures. A positive PCR result makes the presence of CNS IA highly likely and should prompt adequate therapeutic measures. Additional prospective clinical trials encompassing an even larger number of patients with proven and probable CNS IA should validate this finding.
